# Breaking the vicious cycle: a bibliometric and visual analysis of the bidirectional relationship between pruritus and negative emotions

**DOI:** 10.3389/fpsyt.2026.1883590

**Published:** 2026-07-30

**Authors:** Shuman Yu, Siying Qu, Donghai Wu, Yutian Chen, Yixiang Wang, Shuting Zhou, Haiju Sun, Xiaoyu Li

**Affiliations:** 1 The Third Affiliated Hospital of Zhejiang Chinese Medical University (Zhongshan Hospital of Zhejiang Province), Hangzhou, Zhejiang, China; 2 Key Laboratory of Acupuncture and Neurology of Zhejiang Province, The Third Clinical Medical College, Zhejiang Chinese Medical University, Hangzhou, Zhejiang, China; 3 The First Affiliated Hospital of Zhejiang Chinese Medical University (Zhejiang Provincial Hospital of Traditional Chinese Medicine), Hangzhou, Zhejiang, China

**Keywords:** bibliometric analysis, CiteSpace, negative emotions, pruritus, VOSviewer

## Abstract

**Background:**

Pruritus is a common skin symptom, frequently associated with substantial psychological burden. Recent advances in neuroscience and clinical practice suggest a clinically significant reciprocal association between chronic pruritus and negative emotions (e.g., anxiety and depression), moving beyond simple models of causality. Despite increasing research interest, this interdisciplinary field still lacks a comprehensive bibliometric overview. Therefore, this study aims to systematically visualize the research landscape and identify emerging hotspots, underexplored areas, and translational priorities regarding the pruritus-emotion interplay.

**Methods:**

Literature data were retrieved from the Web of Science Core Collection (WoSCC) and supplemented with targeted PubMed clinical trials published from January 1, 1980, to May 31, 2026. WoSCC records served as the main bibliometric dataset, whereas PubMed clinical trials were screened according to predefined eligibility criteria and used as a supplementary clinical-trial dataset. The final included records and records excluded after full-text assessment were verified by a second reviewer. Advanced bibliometric tools, including VOSviewer, CiteSpace, Charticulator and Bibliometrix were used to evaluate collaboration networks, co-citation relationships, and keyword evolution.

**Results:**

A total of 2549 publications from WoSCC and 46 clinical trials from PubMed were included. The United States exhibited absolute advantages in publication volume, citation influence, and international cooperation. The leading institutions were mainly located in North America and Europe, including the University of Toronto, University of Miami, and Wroclaw Medical University. Szepietowski J.C., Yosipovitch G., and Ständer S. were the most productive authors, while Yosipovitch G. and Silverberg J.I. occupied central positions in the co-citation network. The supplementary PubMed clinical trial analysis indicated that targeted pharmacological therapies were more frequently represented than psychological, behavioral, or integrative interventions. The integrated findings identified five interrelated themes: anxiety and depression as the principal emotional constructs, the crucial implementation of multidimensional patient-reported outcome measures (PROMs), sleep and cognitive amplifiers, targeted and integrative care, and skin-brain axis mechanisms.

**Conclusion:**

This bibliometric analysis provides a structured overview of the research landscape on pruritus and negative emotions. By revealing emerging trends and future research priorities, this study serves as a valuable reference for scholars and clinicians exploring interdisciplinary interventions to break this vicious cycle.

## 1 Introduction

Pruritus, commonly known as itch, is a distressing somatosensory experience that triggers a profound desire to scratch, significantly impairing patients’ well-being (1). Epidemiological studies indicate that pruritus ranks among the top 50 most prevalent global health conditions, with a notably higher incidence in Asian and African American populations, particularly among older adults and women (2). Clinically, pruritus is categorized into acute and chronic forms, with the latter defined as persisting for over six weeks (3). Extensive research has demonstrated that chronic pruritus imposes a formidable disease burden, comparable in severity to that of chronic pain (4). Affected individuals frequently endure relentless physical discomfort and severe sleep disruption, which collectively diminish their quality of life and invariably precipitate serious psychological distress (5, 6).

Negative emotions encompass a spectrum of unpleasant affective experiences, such as anxiety, worry, depression, fear, anger, guilt, and shame (7, 8). Crucially, these subjective affective states should be distinguished from clinically defined psychiatric disorders, which require a threshold of clinically significant functional impairment (9). Nonetheless, when persistent or intense, negative emotions can severely impair well-being, sleep, social functioning, and overall quality of life (10). In chronic disease contexts, anxiety and depressive symptoms are particularly salient, as they frequently coexist with physical ailments and exacerbate the cumulative disease burden (11). Additionally, self-conscious emotions such as guilt and shame contribute significantly to symptom-related distress and interpersonal functioning in clinical populations (12).

Accumulating clinical, epidemiological, and experimental evidence suggests a clinically relevant, reciprocal association between chronic pruritus and emotional distress, particularly anxiety and depressive symptoms, although the precise causal pathways remain incompletely understood. On the one hand, psychological distress may initiate, perpetuate, or aggravate pruritus. In certain patients, it contributes substantially to psychogenic pruritus, also known as functional itch disorder (FID). Recognizing this, the International Forum for the Study of Itch (IFSI) categorizes psychiatric/psychosomatic pruritus as a distinct etiology (13). Furthermore, diagnostic criteria proposed by the French Psychodermatology Group (FPDG) for FID emphasize that psychological factors are closely related to the initiation, severity, exacerbation or persistence of pruritus (14). Mechanistically, sustained depressive or anxious states may lower the central perceptual threshold for pruritus, thereby amplifying the sensory experience (15). On the other hand, chronic pruritus is consistently associated with increased emotional burden. Recent evidence from a European multicenter study showed that dermatological patients with pruritus reported significantly higher levels of stress, anxiety, and depression than patients without pruritus (16). One hypothesized reason for this correlation is that impaired hypothalamic-pituitary-adrenal (HPA) axis function in individuals with chronic pruritus may contribute to emotional dysregulation and heightened negative emotions, including anxiety and depressive states (17). In addition to neuroimmune and neuroendocrine pathways, a psychodermatological perspective places chronic pruritus within a broader biopsychosocial context, in which dermatological and sensory mechanisms interact with psychological, behavioral, and social factors (5, 18). Within this framework, cognitive and affective processes such as symptom focused attention, worry, rumination, catastrophic appraisal, and illness perception, may increase symptom salience and reinforce the itch-scratch cycle (18, 19). Concurrently, shame, embarrassment, social avoidance, and dysfunctional metacognitive beliefs may further maintain emotional distress and broaden the psychological burden associated with chronic pruritus (20, 21). Importantly, while therapeutic observations indicate that reductions in pruritus frequently coincide with improvements in psychological outcomes (22, 23), these concurrent improvements should not be strictly interpreted as definitive evidence of unidirectional causality, but rather as indicative of a complex, interwoven relationship.

Building upon this bidirectional framework, the present study employs bibliometric and visual methods to clarify how research on pruritus and negative emotions has been organized and how its thematic focus has evolved. Previous conventional reviews have provided vital clinical and mechanistic insights into this comorbidity (5, 24). However, because this literature spans dermatology, psychiatry, psychology, neuroscience, and immunology, conventional reviews alone may not fully capture its overall intellectual structure, collaboration patterns, and thematic development. Although previous bibliometric studies have mapped related areas, including pain-related research, dermatological research, and psychology or mental health topics (25–27), the specific intersection between pruritus and affective distress has not been systematically visualized. This gap is clinically relevant because pruritus and emotional dysregulation may be interrelated and mutually reinforcing. Bibliometric and visual analyses can therefore complement existing reviews by providing a more comprehensive, intuitive, and data driven field level overview of research development, dominant themes, and underexplored directions (28). Finally, while this study adopted a broad conceptual scope for negative emotions, anxiety and depressive symptoms emerged as the most heavily represented constructs in the bibliometric dataset. Consequently, they are discussed in greater depth, while other negative emotions are highlighted as crucial underexplored directions for future research.

## 2 Materials and methods

### 2.1 Study design and data sources

**Figure 1:**
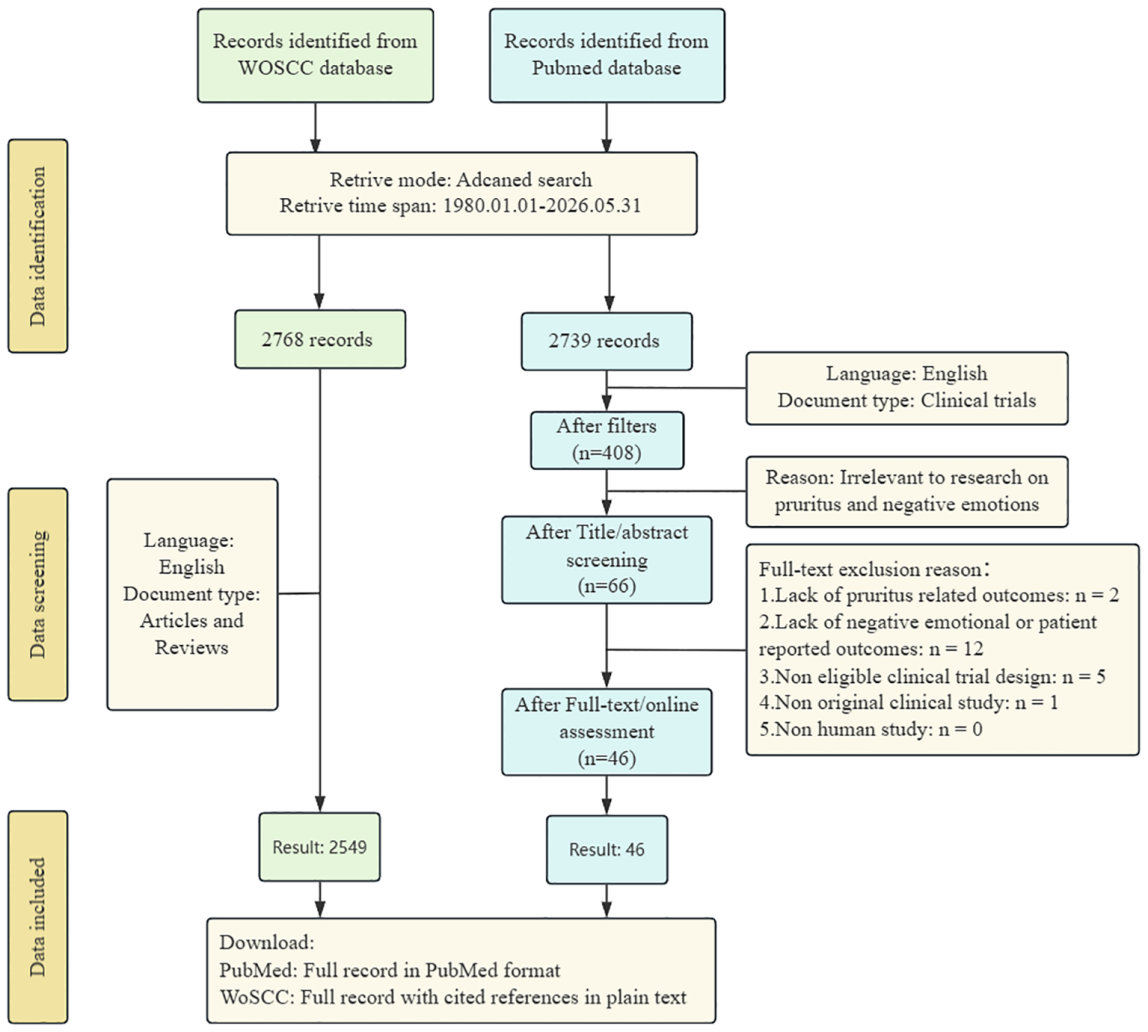
The Flow chart of scientometric analysis.

To ensure a consistent and reproducible bibliometric analysis, this study was based primarily on Web of Science Core Collection (WoSCC) records, supplemented by a targeted PubMed clinical-trial sub-analysis. WoSCC was selected as the primary data source because it is a widely used, high quality multidisciplinary citation database that supports the full bibliometric workflow from literature retrieval to research output evaluation (29). It provides standardized citation metadata, including author information, affiliations, abstracts, references, and cited references, which are essential for co-citation analysis, collaboration network mapping, and citation burst detection (30). PubMed was used as a supplementary source to provide an intervention-oriented view of clinical trials relevant to pruritus and negative emotions. As the flagship biomedical database of the National Library of Medicine, it offers a comprehensive index of clinical research literature and specialized filters for study design (31). Consequently, WoSCC provides a broad literature perspective, while PubMed added insights into clinical trial interventions, supporting a more contextual interpretation of the field. To maintain the consistency of citation metadata and ensure compatibility with the planned co-citation, collaboration, and burst analyses, other databases such as Scopus, Embase, PsycINFO, and CINAHL were not included. Although we carefully reviewed the WoSCC search output, focusing on representative records in psychology, psychiatry, and psychosomatic medicine to minimize the risk of omitting relevant studies, the exclusion of these additional databases may still inherently limit the retrieval of certain specialized nursing or allied health literature. Both databases were searched on June 23, 2026. The complete workflow of literature retrieval, record processing, and inclusion is shown in **Figure 1**.

### 2.2 WoSCC bibliometric dataset construction

The WoSCC search covered publications from January 1, 1980, to May 31, 2026. The search was restricted to English-language articles and reviews. The search strategy was defined as follows: TS = (“pruritus” OR “itching” OR “pruritis” OR “itch” OR “scratchiness”) AND TS = (“anxiety” OR “worry” OR “depression” OR “fear” OR “anger” OR “hostility” OR “guilt” OR “shame” OR “negative emotions” OR “psychological distress” OR “emotional distress” OR “stress” OR “emotion regulation” OR “emotional dysregulation” OR “affective symptoms” OR “mood symptoms” OR “rumination” OR “catastrophizing”).

After database filtering, the retrieved records were checked for duplicate entries. The WoSCC dataset was defined as a bibliometric corpus based on the prespecified search strategy and database filters, without additional manual title/abstract or full-text screening. The final WoSCC records were exported in plain text format with full records and cited references for subsequent bibliometric and visualization analyses.

### 2.3 PubMed clinical trial dataset and screening

The PubMed search used the same time span and English-language restriction as the WoSCC search, and the publication type was limited to clinical trials. The search strategy was as follows: (“pruritus”[Title/Abstract] OR “itching”[Title/Abstract] OR “pruritis”[Title/Abstract] OR “itch”[Title/Abstract] OR “scratchiness”[Title/Abstract]) AND (“anxiety”[Title/Abstract] OR “worry”[Title/Abstract] OR “depression”[Title/Abstract] OR “fear”[Title/Abstract] OR “anger”[Title/Abstract] OR “hostility”[Title/Abstract] OR “guilt”[Title/Abstract] OR “shame”[Title/Abstract] OR “negative emotions”[Title/Abstract] OR “psychological distress”[Title/Abstract] OR “emotional distress”[Title/Abstract] OR “stress”[Title/Abstract] OR “emotion regulation”[Title/Abstract] OR “emotional dysregulation”[Title/Abstract] OR “affective symptoms”[Title/Abstract] OR “mood symptoms”[Title/Abstract] OR “rumination”[Title/Abstract] OR “catastrophizing”[Title/Abstract]). Because the PubMed dataset was analyzed separately to evaluate clinical interventions and was not merged with the WoSCC dataset, cross-database duplicate removal was not performed.

To ensure rigorous study selection, two researchers collaboratively conducted the screening process based on predefined eligibility criteria. Initially, titles and abstracts were screened, followed by a comprehensive cross-verification of all included and excluded full-text records by the second reviewer, with documented reasons for exclusion. Any discrepancies regarding study eligibility were resolved through consensus discussion. Studies were deemed eligible if they: (1) were clinical trials involving human participants; (2) investigated pruritus or pruritus-related disease burden; (3) reported negative emotions, anxiety, depression, psychological distress, sleep disturbance, quality of life, or related patient-reported outcomes; and (4) provided clinical information on interventions that relate to the connection between pruritus and negative emotions. Any inconsistencies in the screening process were resolved through discussion. Because the screening process involved initial screening followed by reviewer verification rather than two fully independent screening decisions for all records before consensus discussion, Cohen’s kappa was not calculated. Eligible records were exported in PubMed format for supplementary keyword co-occurrence analysis.

### 2.4 Visualized analysis

All analytical procedures were conducted using VOSviewer 1.6.20, CiteSpace 6.4.R1, Charticulator, and the Bibliometrix package in the R environment 4.4.1.

VOSviewer, a specialized program for bibliometric analysis, enables researchers to visualize and cluster scientific literature (32, 33). In VOSviewer, nodes show countries, institutions, authors or keywords. The size of network nodes corresponds directly to term frequency, where increased dimensions signify higher occurrence rates in the analysis (34). In the present study, VOSviewer was used to construct institutional collaboration, author collaboration, author co-citation, and keyword co-occurrence networks. To maintain clear network visualization while preserving the major structural information of the dataset, threshold values were predefined for each analysis. These thresholds were selected after preliminary testing to balance network interpretability with preservation of the main structural features of the dataset. Specifically, the thresholds were 13 publications for institutions, 7 publications for authors in collaboration analysis, 42 citations for co-cited authors, 31 occurrences for WoSCC keywords, and 4 occurrences for PubMed keywords. The number of nodes before and after thresholding is provided in **Supplementary Table 1**. For the VOSviewer keyword co-occurrence map, keyword clusters were generated automatically based on co-occurrence patterns, and thematic labels were assigned by the authors according to keyword composition, keyword occurrence, total link strength (TLS), semantic coherence, and representative references. To reduce term fragmentation, keyword terms were standardized before co-occurrence analysis using a manually constructed VOSviewer thesaurus file. This process covered synonyms, spelling variants, and abbreviations. Clinically distinct concepts were not merged to avoid artificially altering the keyword co-occurrence structure. The full thesaurus table is provided in **Supplementary Table 2**. Across all VOSviewer analyses, network normalization was performed using the association strength method, and items that did not meet the preset thresholds were automatically excluded by the software. Citation indicators reported in this study were obtained from the WoSCC records and exported through VOSviewer for bibliometric analysis. These citation values were treated as raw cumulative citation counts and were not normalized by publication year. Therefore, they were interpreted as descriptive indicators of accumulated citation impact rather than direct measures of research quality or current influence, because earlier publications have longer citation windows and may accumulate more citations over time. Author names with diacritics were retained in their original orthographic form as recorded in the WoSCC metadata. After bibliometric processing, spelling variants referring to the same author, including variants with or without diacritical marks, were manually checked against the source records and harmonized for consistent reporting in the manuscript. No systematic normalization of diacritics was applied beyond this manual consolidation.

CiteSpace is a free computing tool designed to identify and illustrate developmental trajectories of scientific research (35). Developed in 2004, this Java-based program helps researchers map knowledge structures and predict research trends in a certain field through temporal network analysis (36). In this study, CiteSpace was used to construct the timeline map of co-cited references and keywords, and to detect bursts patterns of institutions, references, and keywords. For the keyword timeline map, the node type was set to Keyword, and cluster labels were generated automatically by CiteSpace. Burst detection in CiteSpace is based on Kleinberg’s algorithm and identifies sudden increases in the frequency of specific events over time (37). The interpretation of each burst output depends on the selected node type. Specifically, bursts of institution nodes were interpreted as temporal increases in affiliation-linked publication activity, bursts of cited-reference nodes as citation bursts of references, and bursts of keyword nodes as increases in keyword occurrence frequency. All burst outputs included burst strength and the corresponding begin and end years. The analyses were performed with time slicing set from January 1980 to May 2026 and a slice length of 1 year. Node selection was based on the g-index criterion (k = 25), which allowed influential nodes to be retained while keeping the network structure manageable. To simplify the network and improve visual interpretability, Pathfinder and pruning of sliced networks were applied. Unless otherwise specified, the remaining parameters were kept at the default settings.

Charticulator is an interactive visualization tool designed for the creation of custom and reusable chart layouts (38). In this study, we utilized Charticulator to design and generate the international collaboration network diagram.

Bibliometrix is an R package used for bibliometric analysis, which provides tools and functions for analyzing and visualizing bibliographic data (39, 40). In this article, we used Bibliometrix to visualize the temporal distribution of author productivity.

## 3 Results

### 3.1 Annual numbers of publications

**Figure 2:**
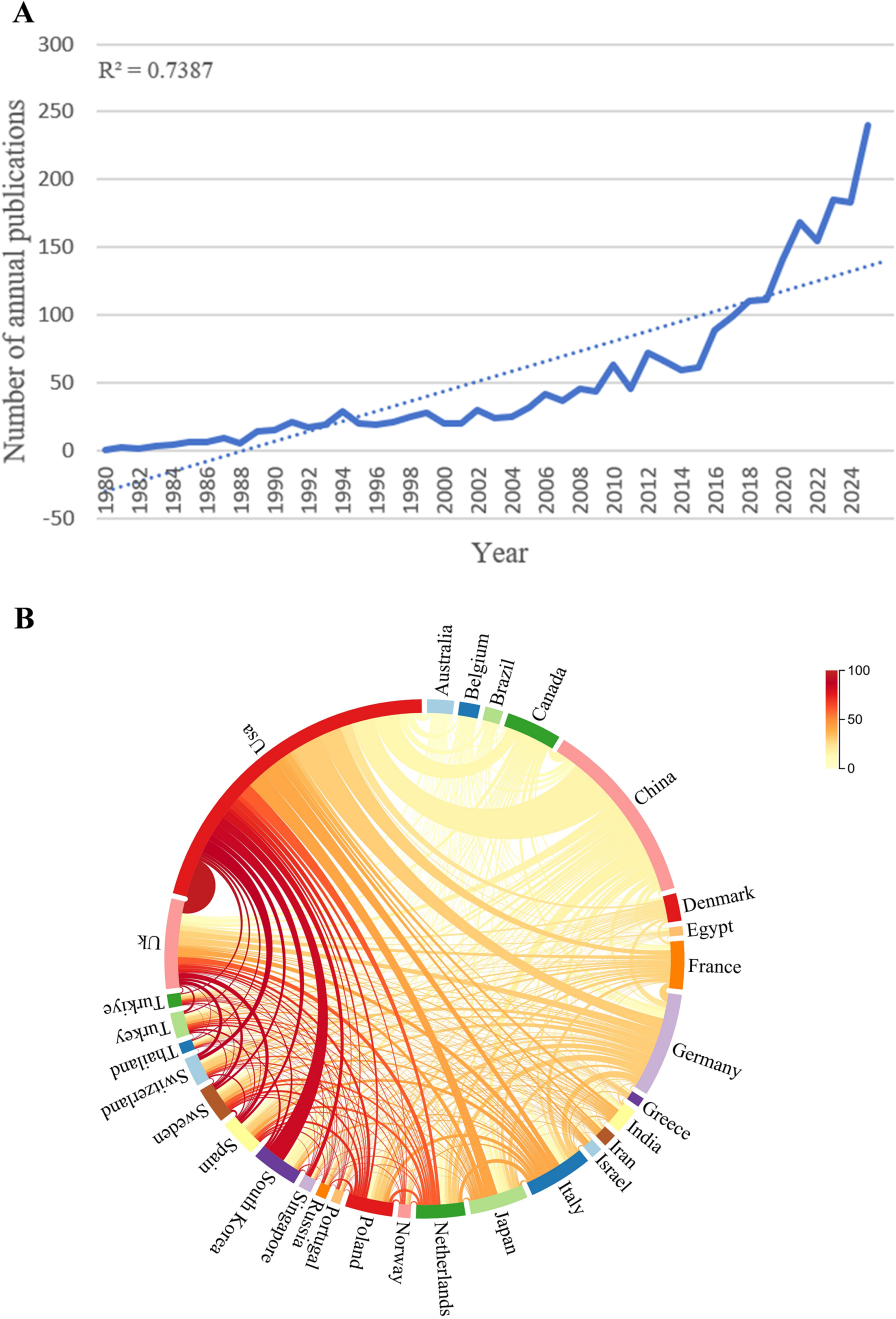
Publication trends and international collaboration in pruritus-negative emotion research based on WoSCC data. **(A)** The number and trend of publication from 1980 to 2025 based on WoSCC data produced by Microsoft Excel 2019. **(B)** The International collaboration network among the top 30 most productive countries/regions based on WoSCC data produced by Charticulator.

A total of 2549 records were retrieved from WoSCC, including 2098 articles and 451 reviews. The annual publication trend in research on pruritus and negative emotions from 1980 to 2025 is presented in **Figure 2A**. Records published in 2026 were not included in this analysis because the database search covered 2026 only up to May 31, and the publication count for that year therefore represented partial-year data. Few publications appeared before 2000, after which output gradually increased during the 2000s and early 2010s. A more pronounced rise was observed after 2016, indicating growing research attention to the relationship between pruritus and negative emotions. The dotted trendline in **Figure 2A** was derived from a simple linear regression model, with publication year as the independent variable and annual publication count as the dependent variable. The R² value of 0.7387 indicates that the model captured a large proportion of the overall increase in annual publication output. This value was used only as a descriptive indicator of the general trend. The annual publication counts are provided in **Supplementary Table 3**.

### 3.2 Distribution of countries or regions analysis

This research topic has generated 2549 publications across 102 countries or regions. **Figure 2B** illustrates the research collaboration patterns among the 30 most productive countries and regions. Each circle represents a country/region. Larger circles indicate more publications, while thicker and darker connecting lines show stronger collaborations. The analysis shows that the research cooperation between China and the United States is particularly strong, and the United States has also demonstrated significant cooperation with the UK. The network connectivity indicators show the United States maintains the most international partnerships, reflected by its highest number of connectivity lines. As shown in **Table 1**, bibliometric indicators confirm that the United States leads in both publication output (759 documents) and citation impact (35474 citations). China follows closely in both metrics, with 423 documents and 7006 citations. Although there are fewer publications in Germany (237 documents), the total number of citations reached 13689. This pattern may reflect differences in research funding, clinical-trial infrastructure, dermatology networks, pharmaceutical investment, and the availability of large epidemiological datasets.

**Table 1:** Top 10 most productive countries or regions in WoSCC.

Rank	Countries or regions	Documents	Total citations
1	USA	759	35474
2	China	423	7006
3	Germany	237	13689
4	UK	205	12338
5	Italy	147	5811
6	Japan	132	5930
7	Canada	126	8315
8	Netherlands	112	5902
9	France	110	8956
10	Poland	108	4089

### 3.3 Distribution of institutions analysis

**Figure 3:**
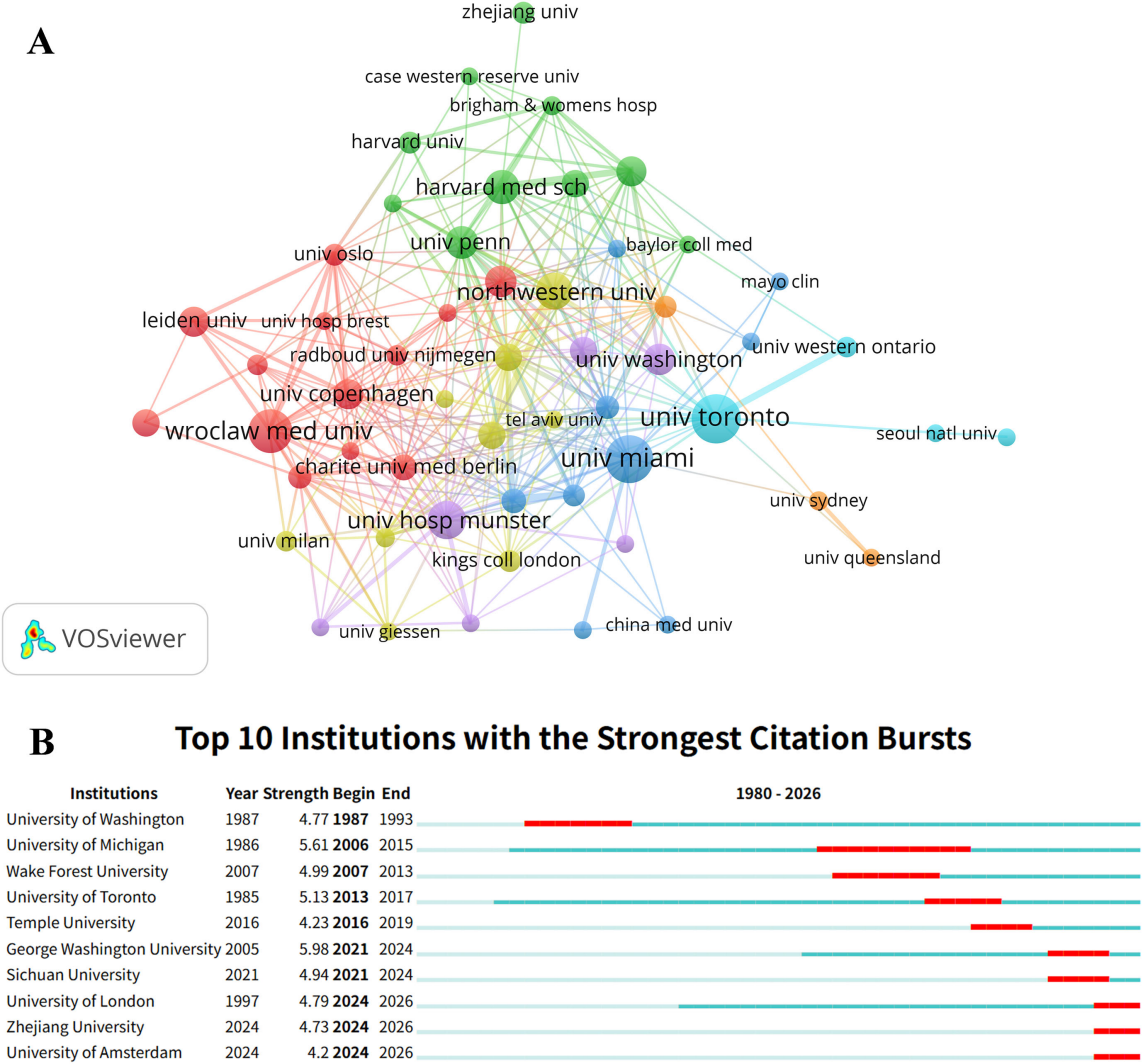
Institutional collaboration network based on WoSCC data for research on the bidirectional relationship between pruritus and negative emotions. **(A)** The institutions’ co-authorship network visualization map produced by VOSviewer. **(B)** The top 10 institutions with the strongest burst in affiliation-linked publication activity from 1980 to 2026 produced by CiteSpace.

The analysis covers contributions from 3634 institutions globally. As presented in **Table 2**, the ten most productive organizations contributed 354 papers, accounting for 13.89% of total publications. **Figure 3A** demonstrates that the University of Toronto (49 documents), University of Miami (48 documents), and Wroclaw Medical University (42 documents) have become leading research institutions. Notably, most of the leading institutions are located in North America and Europe, suggesting that institutional research capacity, access to specialized dermatology or psychosomatic medicine resources, and established collaboration networks may have contributed to their higher publication output. Institutional burst analysis was used to identify research institutions that experience a sudden and significant increase in influence during a specific period of time, thereby helping researchers discover emerging research frontiers. Regarding institutional burst analysis based on affiliation-linked publication activity (**Figure 3B**), the CiteSpace output was generated by selecting “Institution” as the node type and applying the burstness function. The detected bursts indicate periods during which publications affiliated with a given institution increased sharply within the analyzed WoSCC dataset. George Washington University showed the strongest burst strength (5.98), with a burst period from 2021 to 2024. University of Michigan exhibited a relatively long burst period from 2006 to 2015. Notably, University of London, Zhejiang University, and University of Amsterdam have been in an ongoing burst since 2024. In this figure, the “Begin” and “End” years for each institution indicate the beginning and end of the burst period for each institution, defined by a sharp increase in affiliation-linked publication activity.

**Table 2:** Top 10 most productive institutions in WoSCC.

Rank	Organization	Documents	Total citations	Country
1	University of Toronto	49	3544	Canada
2	University of Miami	48	1714	USA
3	Wroclaw Medical University	42	2236	Poland
4	University Hospital Münster	36	2235	Germany
5	Northwestern University	35	3197	USA
6	Harvard Medical School	31	737	USA
7	University of Pennsylvania	30	1211	USA
8	University of California, San Francisco	28	1396	USA
9	University of Washington	28	1511	USA
10	University of Copenhagen	27	1059	Denmark

### 3.4 Authors and co-cited authors analysis

**Figure 4:**
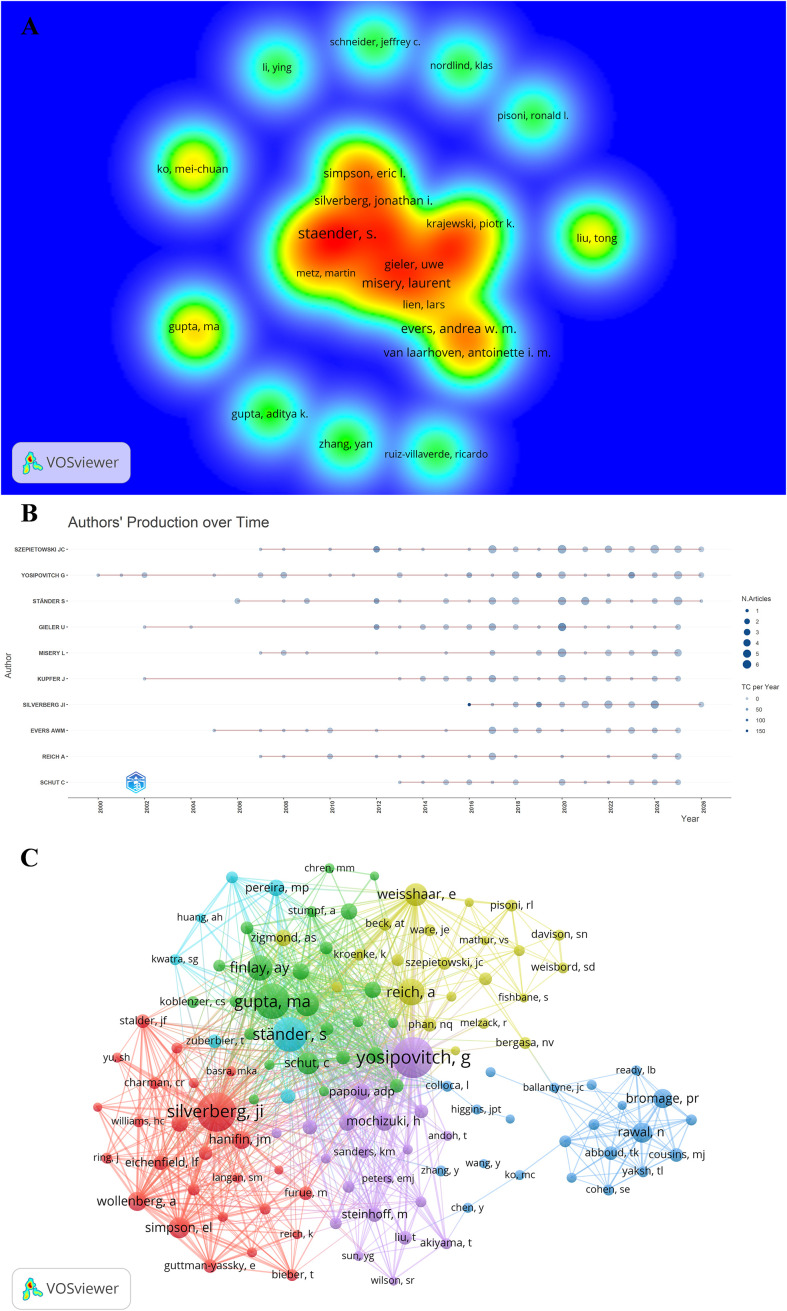
Author collaboration network based on WoSCC data for research on the bidirectional relationship between pruritus and negative emotions. **(A)** The authors’ co-authorship network visualization map produced by VOSviewer. **(B)** The temporal distribution of author productivity produced by Bibliometrix. **(C)** The authors’ co-citation network visualization map produced by VOSviewer.

Among the 2549 publications, a total of 13099 authors are recorded. **Table 3** summarizes the ten leading authors by publication output. Szepietowski J.C. ranked first with 44 publications, followed by Yosipovitch G. with 42 publications and Ständer S. with 41 publications. **Figure 4A** illustrates the most prolific authors and co-authors in the research field of pruritus and negative emotions. In this map, the color gradient from cool to warm tones reflects the density and intensity of research activity within the author’s network. Warm tones such as yellow and red represent a group of authors with high publication volume and close collaboration, who are the core force in this research field. Cooler colors, such as green, indicate less collaboration between authors or reflect a research frontier in its early stages of development. **Figure 4B** presents the publication status of the ten most productive authors at different times. Yosipovitch G. showed the earliest and longest period of activity among the top authors.

**Table 3:** Top 10 most productive authors in WoSCC.

Rank	Author	Documents
1	Szepietowski, J. C.	44
2	Yosipovitch, G.	42
3	Ständer, S.	41
4	Gieler, U.	29
5	Misery, L.	29
6	Kupfer, J.	27
7	Silverberg, J. I.	27
8	Evers, A. W. M.	26
9	Reich, A.	20
10	Schut, C.	20

This study analyzed the co-citation relationships of 61113 authors through network visualization methods. The obtained co-citation network (**Figure 4C**) consists of 122 nodes connected by 4317 edges, forming seven clusters. The TLS is used to measure the degree of connectivity of authors throughout the entire co-citation network. The higher the TLS value, the more extensive the connection between the author’s research and other core researchers in the field (40). **Table 4** lists the ten scholars most frequently co-cited in studies on pruritus and negative emotions. Among them, Yosipovitch G. (480 citations, TLS = 13494) and Silverberg J.I. (442 citations, TLS = 9461) show not only high citation counts but also the strongest network connectivity, fully demonstrating their core position and important influence in pruritus and negative emotions research.

**Table 4:** Top 10 co-cited authors in WoSCC.

Rank	Author	Total citations	Total link strength
1	Yosipovitch, G.	480	13494
2	Silverberg, J. I.	442	9461
3	Gupta, M. A.	385	6140
4	Ständer, S.	367	9897
5	Reich, A.	237	4998
6	Finlay, A. Y.	225	3435
7	Misery, L.	195	4077
8	Weisshaar, E.	181	4808
9	Schut, C.	165	5094
10	Simpson, E. L.	155	5495

### 3.5 Co-cited references analysis

Co-cited reference means that two or more sources are cited together in the same work or across different studies. **Table 5** presents the ten most frequently cited references in citation. Among the top ten highly cited references, Four focusing on scales and assessment tools have collectively established the foundational framework for evaluation in the field of pruritus and negative emotions. Among these, the two most frequently cited are about the Dermatology Life Quality Index (DLQI) ​and the Hospital Anxiety and Depression Scale (HADS), which quantitatively measure the impact of pruritus on quality of life and the potential co-existing negative emotions, respectively (41, 42). The Visual Analogue Scale (VAS), Numerical Rating Scale (NRS), and Verbal Rating Scale (VRS) are commonly used standard tools for quantifying pruritus intensity, with validation studies confirming their reliability and sensitivity, particularly for the VAS (43, 44). Beyond measurement, several top co-cited references directly addressed the psychological and clinical burden of pruritus. In psoriasis, pruritus was highly prevalent and often occurred daily or at night (45). It was also associated with impaired quality of life, stigmatization, stress, and depressive symptoms, while its severity was not fully explained by objective disease severity alone (15, 45). Similarly, research in atopic dermatitis (AD) linked pruritus severity with psychological status, including stress, impaired health-related quality of life, and depressive symptoms (46). Notably, Gupta et al. reported a significant association between pruritus severity and depressive symptoms across psoriasis, AD, and chronic idiopathic urticaria, suggesting that depression may modulate pruritus perception (47). Schneider et al. further showed that psychiatric comorbidity and unmet psychotherapeutic needs were common among patients with chronic pruritus (48).

**Table 5:** Top 10 co-cited references in WoSCC.

Rank	Title	First author	Source	Year	Total citations	IF
1	Dermatology Life Quality Index (DLQI)-a simple practical measure for routine clinical use	Finlay, A. Y.	Clinical and Experimental Dermatology	1994	169	2.8
2	The Hospital Anxiety and Depression Scale	Zigmond, A. S.	Acta Psychiatrica Scandinavica	1983	104	4.7
3	Clinical Classification of Itch: a Position Paper of the International Forum for the Study of Itch	Ständer, S.	Acta Dermato Venereologica	2007	75	3.8
4	The prevalence and clinical characteristics of pruritus among patients with extensive psoriasis	Yosipovitch, G.	The British Journal of Dermatology	2000	75	8.2
5	Depression modulates pruritus perception: a study of pruritus in psoriasis, atopic dermatitis, and chronic idiopathic urticaria.	Gupta, M. A.	Psychosomatic Medicine	1994	66	2.4
6	Assessment of Pruritus Intensity: Prospective Study on Validity and Reliability of the Visual Analogue Scale, Numerical Rating Scale and Verbal Rating Scale in 471 Patients with Chronic Pruritus	Phan, N. Q.	Acta Dermato Venereologica	2012	56	3.8
7	Psychosomatic cofactors and psychiatric comorbidity in patients with chronic itch	Schneider, G.	Clinical and Experimental Dermatology	2006	48	2.8
8	Pruritus is an Important Factor Negatively Influencing the Well-being of Psoriatic Patients	Reich, A.	Acta Dermato Venereologica	2010	46	3.8
9	Visual Analogue Scale: Evaluation of the Instrument for the Assessment of Pruritus	Reich, A.	Acta Dermato Venereologica	2012	45	3.8
10	Relationship between itch and psychological status of patients with atopic dermatitis	Chrostowska−Plak, D.	Journal of the European Academy of Dermatology and Venereology	2013	44	9.2

**Figure 5:**
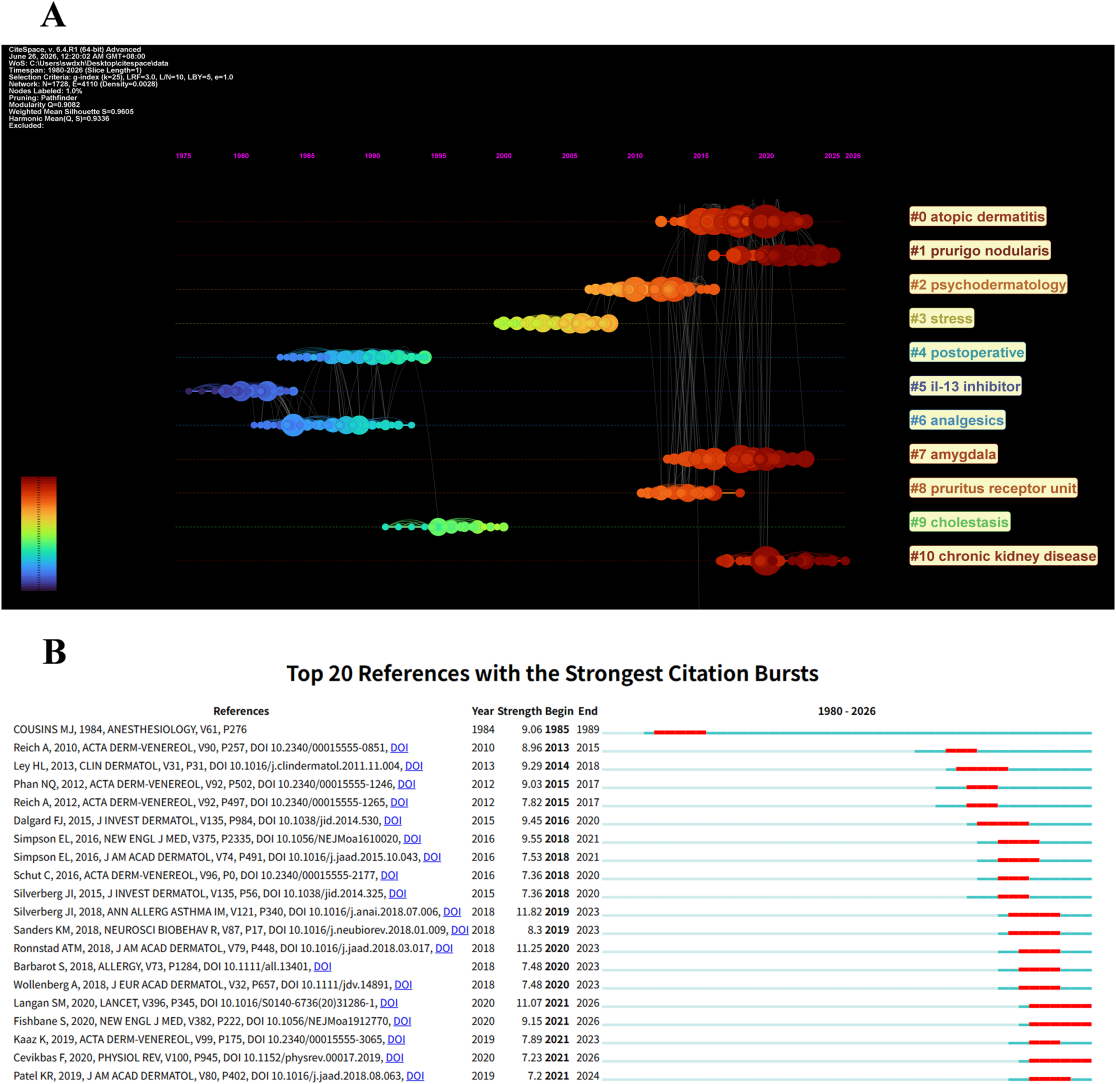
Co-cited references analysis based on WoSCC data for research on the bidirectional relationship between pruritus and negative emotions. **(A)** Co-cited references timeline map produced by CiteSpace. **(B)** The top 20 references with the strongest citation bursts from 1980 to 2026 produced by CiteSpace.

The timeline analysis in **Figure 5A** divides 87190 co-cited references into 11 different clusters, revealing the evolution of research focus over time. The largest grouping is #0 (atopic dermatitis). Previous studies frequently mentioned stress (#3), postoperative (#4), il-13 inhibitor (#5), analgesics (#6), and cholestasis (#9). Since 2010, new research focuses have emerged, including atopic dermatitis (#0), prurigo nodularis (#1), il-13 inhibitor (#5), hidradenitis suppurativa (#6), amygdala (#7), pruritus receptor unit (#8), and chronic kidney disease (#10). However, the latest hotspots including prurigo nodularis (#1) and chronic kidney disease (#10), emerged in 2015 and continue to this day.

Analysis of citation bursts reveals the dynamic progression of intellectual concepts in this domain. **Figure 5B** presents a list of 20 highly cited references. The blue line indicates the publication period of the reference, while the red line denotes a burst of citation frequency. The strongest citation burst, with an intensity of 11.82, was a cross-sectional study of adult AD patients in the United States, which reported that greater AD severity was associated with poorer quality of life and worse mental health, suggesting that worsening pruritus may be associated with greater psychological burden (49). Three publications continue to demonstrate sustained citation bursts in recent years. The review by Langan et al. provided a comprehensive overview of AD, highlighting intense pruritus, psychosocial burden, and mental health comorbidities as important components of disease burden (50). The phase 3 trial by Fishbane et al. reported that difelikefalin significantly reduced pruritus intensity and improved pruritus-related quality of life in hemodialysis patients with moderate-to-severe pruritus, suggesting that chronic pruritus treatment may extend beyond symptom control to broader patient-reported burden (51). The review by Cevikbas and Lerner summarized the physiology and pathophysiology of pruritus, supporting the interpretation of chronic pruritus as a multidimensional condition rather than merely a cutaneous symptom (4). Together, these sustained citation bursts indicate that recent influential literature has primarily strengthened the disease-burden, therapeutic, and mechanistic basis. These foundations provide important entry points for further investigation of the link between pruritus and negative emotions.

### 3.6 Keyword analysis

**Figure 6:**
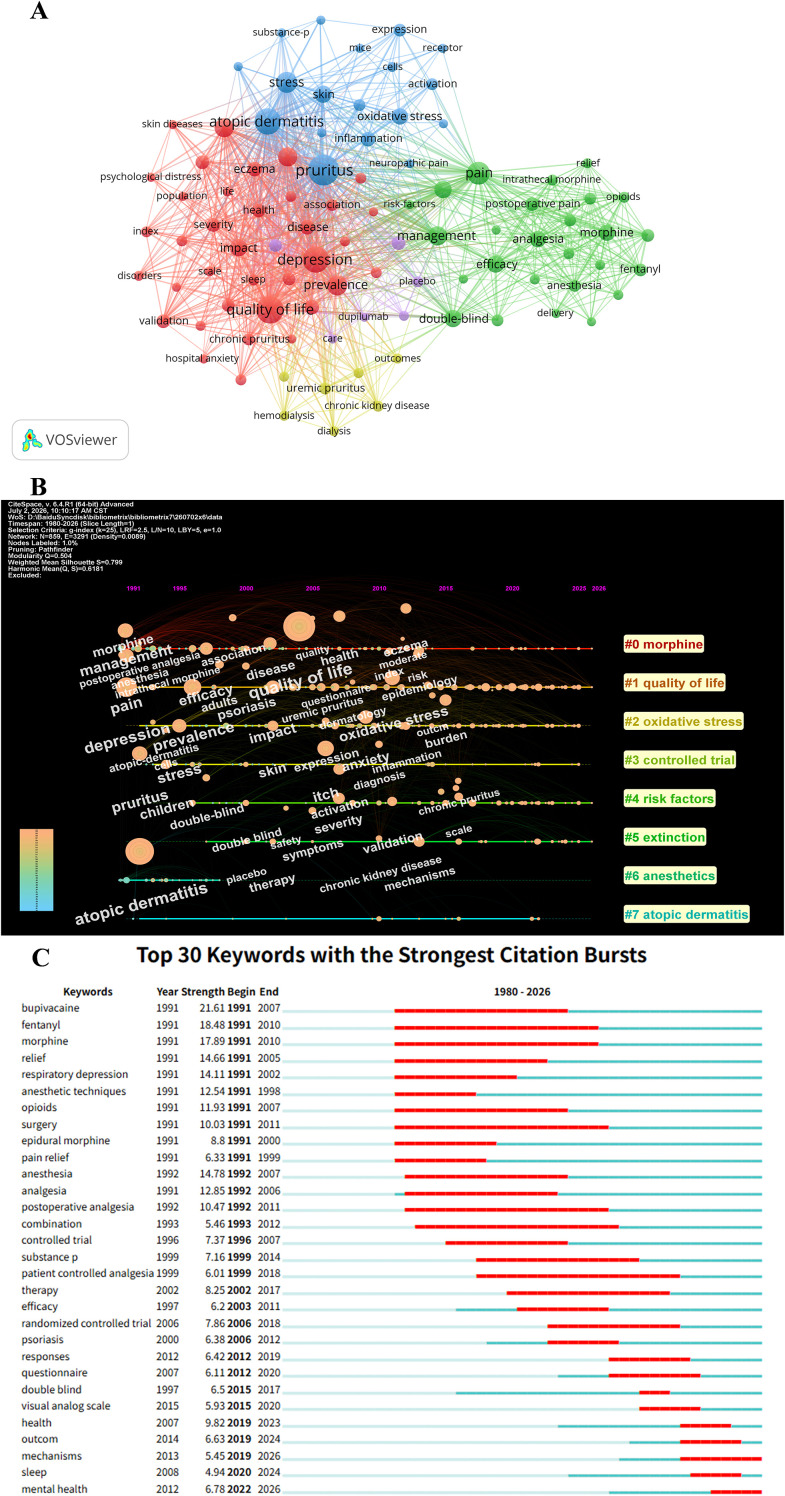
Keyword analysis of research on the bidirectional relationship between pruritus and negative emotions based on WoSCC data. **(A)** The keywords’ co-occurrence network visualization map produced by VOSviewer. **(B)** Keyword timeline map produced by CiteSpace. **(C)** The top 30 keywords with the strongest citation burst from 1980 to 2026 produced by CiteSpace.

Keyword co-occurrence reflects the areas of interest and potential future directions. To ensure that our research is as comprehensive as possible, we use VOSviewer to generate the keyword co-occurrence map (**Figure 6A**) and frequency ranking of keywords (**Table 6**), selecting the “All Keywords” option to incorporate both Author Keywords and Keywords Plus. Within the co-occurrence network, a larger node diameter indicates a higher frequency of the keyword and a greater significance.

**Table 6:** Top 20 most frequent keywords in WoSCC.

Rank	Keyword	Occurrences	Rank	Keyword	Occurrences
1	pruritus	595	11	double-blind	153
2	quality of life	456	12	skin	152
3	depression	408	13	efficacy	152
4	atopic dermatitis	405	14	impact	150
5	pain	289	15	children	145
6	stress	230	16	morphine	143
7	prevalence	212	17	oxidative stress	143
8	psoriasis	202	18	analgesia	137
9	anxiety	201	19	eczema	106
10	management	189	20	disease	106

The keyword co-occurrence map contained 93 nodes and 2,493 links after applying the predefined threshold of 31 occurrences. To make the labeling process more transparent, **Supplementary Table 4** provides the cluster number, cluster label, number of keywords, top 10 keywords, interpretation, and representative references for each cluster. The map identifies five clusters: Cluster 1 (red) mainly captured the clinical burden, assessment, and psychosocial outcomes of pruritus; Cluster 2 (green) was mainly related to pain management, anesthesia, and opioid-associated pruritus; Cluster 3 (blue) reflected inflammatory and neurobiological mechanisms; Cluster 4 (yellow) focused on chronic kidney disease (CKD) associated pruritus; and Cluster 5 (purple) was mainly associated with therapeutic research and clinical care. These clusters help us identify the most relevant themes and subthemes within the research domain and reveal interdisciplinary areas through co-occurrence maps. The 20 most frequently occurring keywords are summarized in **Table 6**. Among them, atopic dermatitis (405 occurrences), psoriasis (202 occurrences) and eczema (106 occurrences) are the most studied skin conditions, which may indicate that pruritus and negative emotions often coexist in these three diseases. In addition, two negative emotions emerge among the top 20 keywords, which are depression (408 occurrences) and anxiety (201 occurrences), indicating that they have received the most attention in research on interactions with pruritus.

To further examine the temporal evolution of research hotspots, a keyword timeline map was generated (**Figure 6B**). The network showed a clear and interpretable clustering structure, with a modularity Q value of 0.504 and a weighted mean silhouette score of 0.799. Earlier studies were mainly concentrated in clusters related to morphine (#0) and anesthetics (#6), including postoperative analgesia, anesthesia, and intrathecal morphine, suggesting that early research was largely embedded in opioid associated and procedure related pruritus. In recent years, the active clusters have shifted toward quality of life (#1), oxidative stress (#2), risk factors (#4), and extinction (#5). The quality of life cluster, represented by depression, questionnaire, and burden, reflects the increasing emphasis on patient reported burden and emotional outcomes. The oxidative stress cluster, including inflammation, stress, and mechanisms, indicates that biological explanations of pruritus have become more prominent. The risk factors cluster suggests continued attention to clinical susceptibility and disease related determinants. The extinction cluster, involving conditioning and nocebo, indicates growing interest in how learning, expectation, and central processing may influence pruritus experience. Overall, the timeline map suggests a gradual shift from analgesia and opioid associated pruritus toward patient burden, emotional outcomes, biological mechanisms, risk stratification, and cognitive neurobiological interpretation.

Another crucial sign of study frontiers, hotspots and the rising trends over time is the strength of the keywords’ burst (**Figure 6C**). A keyword burst occurs when a specific keyword shows a sharp rise in its occurrence frequency during a certain period, indicating a surge of academic interest. Correspondingly, the “strength” of a burst quantifies the intensity of this increase, with higher values representing more focused research attention. This analysis was conducted using CiteSpace, processing both Author Keywords and Keywords Plus to capture a complete spectrum of emerging trends. The strongest early bursts were mainly related to anesthesia and opioid associated pruritus, corresponding to the VOSviewer cluster on pain management and analgesia-related pruritus. Bupivacaine showed the highest burst strength (21.61), followed by fentanyl (18.48) and morphine (17.89). Later bursts, including therapy, randomized controlled trial, questionnaire, and visual analog scale, reflected increasing attention to clinical evaluation and symptom measurement. More recent burst keywords included health, outcome, mechanisms, sleep, and mental health, with mechanisms and mental health continuing until 2026. This pattern is consistent with the keyword timeline map, suggesting that research attention has gradually expanded from analgesia and opioid associated pruritus toward patient centered outcomes, biological mechanisms, sleep disturbance, mental health, and cognitive dimensions of pruritus.

### 3.7 Supplementary analysis of PubMed clinical trials

To further characterize clinical intervention research related to pruritus and negative emotions, we conducted a supplementary PubMed analysis of eligible clinical trials.

The PubMed search initially retrieved 408 clinical trial records. After title and abstract screening, 342 records were excluded because they did not meet the predefined eligibility criteria. The full texts of 66 records were assessed for eligibility, and 20 were excluded after full-text assessment. The main reasons for exclusion were lack of pruritus-related outcomes (n=2), lack of negative emotional or patient-reported outcomes (n=12), non-eligible clinical trial design (n=5), non-original clinical study (n=1), non-human study (n=0). Finally, 46 clinical trials were included in the supplementary PubMed clinical trial analysis, and keyword co-occurrence analysis was performed using VOSviewer.

**Figure 7:**
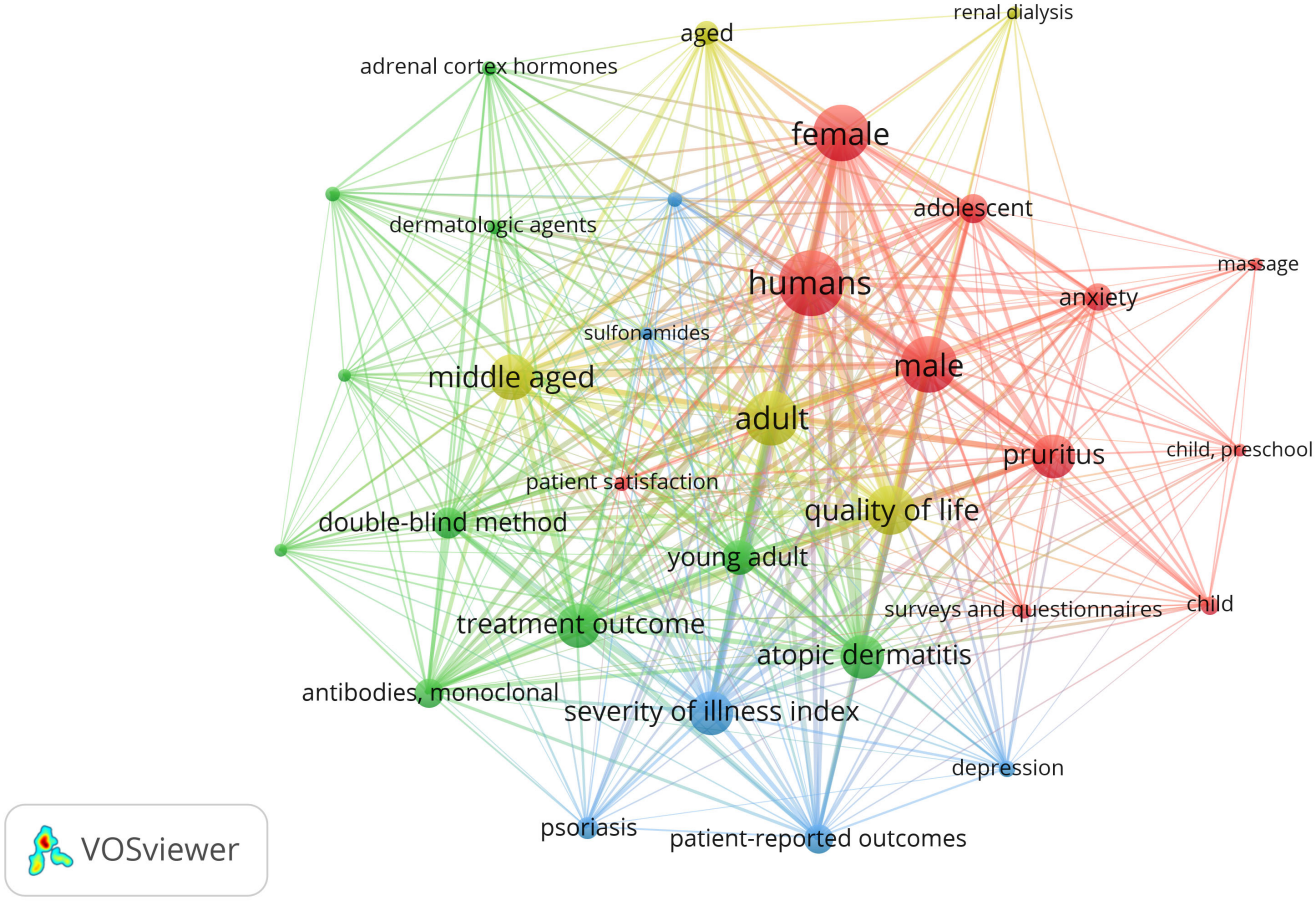
The keywords’ co-occurrence network visualization map based on PubMed clinical-trial dataset produced by VOSviewer.

Through the keyword co-occurrence clustering shown in **Figure 7**, this study identified four main research clusters: Cluster 1 (red) centers on pruritus-related symptom burden, anxiety, and patients’ perceptions of outcomes, with a particular focus on pediatric and adolescent populations; Cluster 2 (green) addresses atopic dermatitis (AD) and clinical trial methodology with targeted therapies; Cluster 3 (blue) focuses on psoriasis, depression, and patient-reported outcome measures (PROMs); Cluster 4 (yellow) pertains to pruritus associated with CKD, quality of life, and older adult populations. **Table 7** lists the ranking of keywords with high frequency, providing supplementary information for further understanding the most prominent terms within this clinical trial dataset.

**Table 7:** Top 20 most frequent keywords in the PubMed clinical-trial dataset.

Rank	Keyword	Occurrences	Rank	Keyword	Occurrences
1	humans	45	11	young adult	18
2	female	37	12	double-blind method	15
3	male	36	13	patient-reported outcomes	13
4	adult	35	14	antibodies, monoclonal	13
5	quality of life	29	15	adolescent	13
6	middle aged	27	16	anxiety	12
7	pruritus	24	17	aged	10
8	atopic dermatitis	24	18	psoriasis	9
9	severity of illness index	24	19	child	7
10	treatment outcome	24	20	depression	6

Based on the clustering results and high-frequency keyword ranking, this study summarizes several research hotspots in clinical trials in this field. Specifically, it includes the age and gender heterogeneity of the research subjects reflected by keywords such as “adolescent” (13 occurrences), “adult” (35 occurrences), “middle aged” (27 occurrences), “aged” (10 occurrences), “female” (37 occurrences), and “male” (36 occurrences); typical key disease models represented by “atopic dermatitis” (24 occurrences) and “psoriasis” (9 occurrences); core negative emotions such as “depression” (6 occurrences) and “anxiety” (12 occurrences); patient-centered assessment with a focus on “patient-reported outcomes” (13 occurrences); and targeted therapy strategies indicated by “antibodies, monoclonal” (13 occurrences).

Beyond keyword co-occurrence, the included PubMed clinical trial records were narratively grouped according to intervention type and outcome domain. Most clinical trials focused on inflammatory skin diseases, particularly AD, psoriasis, and prurigo nodularis (PN), with additional evidence from CKD associated pruritus and burn-related pruritus. Targeted pharmacological therapies represented the most prominent intervention direction, especially biologics such as dupilumab and nemolizumab, and Janus kinase (JAK) inhibitors such as upadacitinib and abrocitinib. In contrast, fewer trials evaluated non-drug or integrative approaches, including cognitive behavioral therapy (CBT), relaxation interventions, acupuncture, massage therapy, structured educational interventions, and support groups. These interventions were more directly related to anxiety, sleep disturbance, patient perception, or psychosocial burden. Across the included trials, pruritus was generally assessed together with dermatology-specific quality of life, sleep disturbance, anxiety, depression, or broader patient-reported outcomes. However, direct emotional outcomes were not measured uniformly. Some studies used dedicated psychological instruments, whereas others relied mainly on quality of life measures such as the DLQI rather than anxiety or depression specific scales. Overall, the supplementary PubMed clinical-trial dataset suggests that clinical research has increasingly moved from pruritus intensity alone toward multidimensional patient-centered outcomes, but the number of eligible studies remains limited.

## 4 Discussion

No previous bibliometric analysis has been applied to the studies exploring the bidirectional relationship between negative emotions and pruritus. This study covers publications from 1980 to 2026, providing a broad overview of the research landscape across nearly five decades. By integrating data from two complementary databases and employing multiple visualization tools, we not only identified major research hotspots but also highlighted several underexplored but clinically relevant areas that merit future investigation.

### 4.1 Global trends

Publication trends showed that research on pruritus and negative emotions remained limited before 2000, increased gradually during the 2000s and early 2010s, and rose more markedly after 2016. This pattern indicates that the relationship between pruritus and negative emotions has attracted rapidly growing attention in recent years. Among the 102 countries analyzed, the United States demonstrates absolute field dominance, accounting for the highest publication output and extensive international cooperation. The most productive institutions were mainly located in North America and Europe, including the University of Toronto, University of Miami, and Wroclaw Medical University. These findings suggest that the current knowledge structure of pruritus and negative emotion research is shaped not only by academic interest, but may also be influenced by broader differences in research resources, clinical research platforms, and publication visibility across regions. The present study shows that top scholars such as Szepietowski J.C., Yosipovitch, G., and Ständer S., have promoted significant developments in this field. Their research involves the clinical classification of chronic pruritus, disease burden in AD and psoriasis, psychosomatic comorbidity, and the interaction between pruritus and emotional symptoms.

### 4.2 Research hotspots, thematic shifts, and underexplored areas

By synthesizing evidence from keyword co-occurrence, co-cited references, and the supplementary PubMed clinical trial analysis, we identified several core research hotspots alongside notable knowledge gaps. These findings are discussed below under five thematic priorities that emerged from our data: the predominance of anxiety and depression as emotional constructs, the shift toward multidimensional patient-reported outcomes, the role of sleep and cognitive amplifiers, the translational imbalance between pharmacological and integrated care, and mechanistic insights from a skin-brain axis perspective.

#### 4.2.1 Anxiety and depression as dominant emotional constructs

Among the various negative affective states captured in the literature, anxiety and depression stand out as the most frequently examined emotional constructs. This dominance may partly reflect their high clinical relevance, but it may also be driven by the widespread availability of standardized instruments including HADS, the Generalized Anxiety Disorder-7 (GAD-7), the Patient Health Questionnaire-9 (PHQ-9), and DLQI-related emotional domains. Consistent with this measurement pattern, the high co-occurrence frequencies of anxiety and depression in the keyword network, together with their sustained presence in citation bursts, indicate that researchers have consistently prioritized these two emotional dimensions when investigating the psychological correlates of chronic pruritus. This focus is clinically justified, given that anxiety and depressive symptoms are not only common in dermatological populations but also consistently associated with greater disease burden, unmet psychotherapeutic needs, and reduced quality of life (48, 52). However, other affective states, including shame, guilt, anger, embarrassment, and disgust, remain less visible despite their potential relevance to the psychosocial burden of chronic pruritus. Their underrepresentation may partly stem from the lack of brief, dermatology-specific assessment tools, but it also points to a conceptual gap in how the psychological burden of chronic pruritus is currently framed. Future studies could benefit from broadening the emotional scope beyond anxiety and depression, particularly those that may influence patient experience, coping strategies, and the psychosocial burden of chronic pruritus.

#### 4.2.2 From pruritus intensity measures to multidimensional PROMs

The bibliometric findings suggest a temporal shift in assessment emphasis from pruritus intensity measures toward multidimensional patient-reported outcomes. In the co-cited reference analysis, highly cited studies on the VAS, NRS, and VRS indicate that pruritus severity assessment formed an important methodological basis of this field. More recent signals from the keyword timeline map and citation bursts, including quality of life, questionnaire, outcome, health, sleep, and mental health, suggest that research attention has gradually expanded toward patients’ broader self-reported burden. This shift reflects the growing recognition that the burden of pruritus includes emotional distress, sleep disturbance, cognitive responses, functional impairment, and quality of life, most of which depend on patient’s self-report (18, 19, 53).

Recent clinical and methodological studies further support this pattern. A study on the core outcome areas of chronic skin diseases showed that patients valued outcomes beyond clinician-assessed signs, with pruritus, anxiety, and depression identified as important areas of chronic skin disease (54). Another study further showed that the Patient-Reported Outcome Measurement Information System (PROMIS), a general patient-reported outcome measurement system, can capture physical, emotional, and social effects of chronic skin diseases, including pruritus intensity, anxiety, depression, social satisfaction, and social isolation (55). Evidence from CKD-associated pruritus also supports the need for multidimensional assessment. A systematic review found substantial heterogeneity in patient reported outcomes and measures used in CKD-associated pruritus studies, covering pruritus, quality of life, sleep disturbance, and emotional distress (56). This indicates that patient reported assessment has expanded beyond symptom intensity, but also that greater standardization is needed across disease contexts.

Current PROM frameworks may still underrepresent cognitive and emotional processes that influence how patients perceive and respond to pruritus. Psychological research on chronic pruritus suggests that attention, affect, expectancies, and worrying may shape pruritus experience and scratching behavior (18, 19). Broader chronic disease research indicates that emotion regulation difficulties and maladaptive metacognitive beliefs are relevant to psychological adjustment (21, 57). In addition, visible or socially stigmatizing skin conditions may involve shame, self-disgust, or body-related distress, which can further affect quality of life and social functioning (20). These dimensions remain less visible than pruritus intensity, sleep, quality of life, anxiety, and depression in current assessment practices.

Therefore, future studies should adopt multidimensional PROMs, according to the research question rather than relying only on pruritus intensity or dermatology-specific quality of life. In practical terms, pruritus severity and burden may be assessed using the NRS, VAS, or 5-D Itch Scale (43, 44, 58); quality of life using the DLQI or Skindex (41, 59); anxiety and depressive symptoms using the HADS, GAD-7, or PHQ-9 (42, 60, 61); and sleep disturbance using the Pittsburgh Sleep Quality Index or PROMIS Sleep Disturbance (55, 62). For studies focusing on the cognitive and emotional dimensions of the pruritus-emotion cycle, additional measures may include the Difficulties in Emotion Regulation Scale for emotion regulation difficulties (63), the Metacognitions Questionnaire-30 or other validated metacognitive assessment tools for metacognitive beliefs (64, 65), and clinically relevant instruments for shame, guilt, or body related distress (12, 20). Such an assessment strategy may help future studies capture not only pruritus severity, but also the emotional and cognitive processes that shape patient burden and symptom persistence.

#### 4.2.3 Clinical and psychological amplifiers of the pruritus-negative emotion cycle

The bibliometric findings also point to sleep disturbance and cognitive processes as clinically relevant but insufficiently developed themes. Recent burst keywords, including sleep and mental health, suggest growing attention to factors that may amplify the cycle between pruritus and negative emotions. Meanwhile, terms in the keyword timeline map related to conditioning, nocebo, and central processing indicate emerging interest in cognitive and perceptual processes, although these processes remain less visible than pruritus intensity, quality of life, anxiety, and depression.

Sleep disturbance should be understood not only as the result of chronic pruritus, but also as a clinical amplifier of the cycle between pruritus and negative emotions (5, 66). Persistent pruritus, especially at night, usually disrupts sleep initiation and maintenance (67). Diurnal changes in epidermal barrier function, skin temperature, and neuroendocrine activity may partly contribute to the nocturnal worsening of pruritus (67). Once sleep deprivation or fragmentation occurs, emotional regulation may worsen, increasing the susceptibility to anxiety and depression symptoms in patients already suffering from chronic pruritus (5, 68). At the same time, poor sleep may further damage emotional regulation and may interact with nocturnal scratching, leading to persistent pruritus symptoms (68–70). Therefore, sleep disturbance occupies an intermediate position between nocturnal pruritus, emotional dysregulation, and adverse clinical outcomes.

Beyond sleep disturbance, cognitive and metacognitive processes may also serve as important psychological amplifiers linking pruritus with emotional distress (18, 19, 71). Patients with chronic pruritus may develop persistent worry about symptom recurrence, attentional monitoring of bodily sensations, and beliefs that pruritus-related thoughts are uncontrollable or threatening. These processes may increase symptom salience, maintain anxiety and amplify pruritus perception, thereby reinforcing the cycle between pruritus and negative emotions (18, 19, 64). This interpretation is consistent with psychological research showing that attention, affect, expectancies, helplessness, and worrying can shape pruritus experience and scratching behavior (18, 19). Human experimental evidence further strengthens this cognitive amplifier model. Pruritus can be induced or intensified even without new peripheral stimulation, for example by thinking about pruritus or by exposure to pruritus-related visual cues alone, a phenomenon commonly described as contagious itch or cue-evoked itch, and this response appears particularly relevant in AD (72, 73). This finding is clinically important because it suggests that pruritus is not determined only by peripheral inflammation or skin barrier dysfunction, but can also be amplified by top-down processes such as symptom attention, expectation, memory, and learned associations.

This top-down amplification also has a central neural basis. In patients with AD, functional magnetic resonance imaging (fMRI) evidence indicates that contagious itch involves brain regions related to motor preparation, reward processing, cognitive evaluation, and memory-related processing, including fronto-striatal and memory-related regions (74). These findings help explain how pruritus-related cues may acquire sensory and emotional salience, thereby transforming symptom-focused attention or memory of previous pruritus into real pruritus perception and scratching motivation. Negative emotional states may further intensify this pathway. Stress, anxiety, and depressive symptoms can heighten bodily vigilance, increase the salience of pruritus-related cues, and reduce the perceived controllability of pruritus-related thoughts (18, 19, 75). Importantly, stress may amplify the cycle not only by changing perception, but also by shaping behavior. Acute stress can increase spontaneous scratching in patients with AD even when experimentally evoked pruritus does not increase, suggesting that stress-related scratching may help maintain the itch-scratch cycle independently of proportional changes in subjective pruritus intensity (76).

Direct evidence on pruritus-specific metacognitive processes remains limited. Nevertheless, evidence from physical illness and other psychiatric or psychosomatic conditions suggests that maladaptive metacognitive beliefs may intensify anxiety and depressive symptoms by altering how patients respond to intrusive thoughts, emotional distress, and bodily experiences (21, 77). This evidence gap indicates that metacognitive processes remain insufficiently developed in pruritus research. Existing studies have more often focused on pruritus intensity, sleep, quality of life, anxiety, and depression, whereas pruritus specific metacognitive beliefs, symptom hypervigilance, rumination about pruritus, perceived uncontrollability, cue reactivity, memory-related pruritus processing, and learned scratching responses have rarely been examined. Taken together, sleep disturbance and cognitive or metacognitive processes represent clinically important amplifiers that can worsen emotional distress, maintain symptom attention, reinforce cue-related scratching, increase patient-reported burden, and contribute to poorer clinical outcomes. Future studies should therefore assess these factors more systematically and examine whether targeting sleep, symptom monitoring, cue reactivity, stress-related scratching, rumination, and perceived uncontrollability can reduce the persistence of both pruritus and emotional distress.

#### 4.2.4 Translational shift: targeted biologics and integrative psychodermatological care

Keyword co-occurrence analysis and the supplementary PubMed clinical trial dataset indicate that therapeutic research has become a visible translational hotspot in this field. In the WoSCC keyword network, the therapeutic cluster was mainly associated with clinical care and treatment-related research, while the PubMed clinical trial analysis identified AD, psoriasis, and PN as prominent disease models. Within these clinical trials, targeted pharmacological therapies were more frequently represented, whereas psychological, behavioral, and integrative interventions were less frequently represented despite their clinical relevance. This pattern provides the basis for discussing targeted biologics together with integrated psychodermatological care. In some cases, traditional antihistamines may alleviate pruritus and related emotional distress, but their therapeutic effects are often limited (78). Because chronic pruritus may be sustained by inflammatory, neural, cognitive, emotional, and behavioral processes, treatment should not be limited to pharmacological symptom control (18, 71, 79). For patients with marked anxiety, depressive symptoms, sleep disturbance, persistent symptom monitoring, or compulsive scratching, an integrated approach combining dermatological treatment with psychological and behavioral interventions may be more clinically appropriate (71, 79).

Psychological and behavioral interventions therefore deserve greater attention in this field (80). These approaches target distinct yet interconnected mechanisms, ranging from cognitive reappraisal and stress reduction to behavioral modification and metacognitive regulation, and can be tailored to patients’ predominant symptom profiles. CBT is particularly relevant when pruritus is accompanied by perceived stress, sleep disturbance, depressive symptoms, or repetitive scratching (81). For patients whose pruritus fluctuates with emotional tension or daily stressors, stress management strategies may offer additional value. A psychoeducational program for AD was shown to improve pruritus, disease severity, and several psychosocial outcomes (82). Beyond cognitive and stress-focused approaches, behavioral techniques directly target the scratching response. Habit reversal training and scratch control target the behavioral reinforcement of pruritus by helping patients recognize scratching triggers and replace automatic scratching with competing responses. Habit reversal training has been tested in AD, and review evidence suggests that both habit reversal training and arousal reduction may help relieve chronic pruritus when scratching behavior and physiological arousal contribute to symptom persistence (71, 83). Mindfulness-based approaches, particularly online mindfulness and self-compassion training, may further reduce reactivity to unpleasant bodily sensations and stress-related symptom amplification (84). Acceptance and commitment therapy may further support patients coping with chronic pruritic skin conditions by strengthening psychological flexibility, acceptance, and values-oriented coping, with a family program based on acceptance and commitment therapy shown to improve eczema symptoms and parental self efficacy in children with eczema (85). For patients whose symptom burden is dominated by rumination, perceived uncontrollability, and persistent monitoring of pruritus-related thoughts, metacognitive therapy and detached mindfulness may be considered as exploratory directions (86).

At the service level, integrated psychodermatology clinics may provide a useful care model by combining dermatological treatment, psychological assessment, scratch-control training, sleep management, patient education, and referral for psychotherapy when needed (5, 79, 87). Selected antidepressants such as sertraline or doxepin, and gabapentinoids such as gabapentin or pregabalin, may also be considered in carefully selected patients, particularly when chronic pruritus coexists with depressive symptoms, anxiety, neuropathic features, or sleep disturbance (78, 88, 89).

Alongside these psychological and behavioral approaches, targeted biologics have become an important treatment frontier for pruritic inflammatory skin diseases. Evidence from AD, psoriasis, and PN suggests that biologic treatment can reduce pruritus and may also improve patient-reported outcomes, including sleep disturbance, quality of life, anxiety, and depressive symptoms. For example, dupilumab improved pruritus, sleep, anxiety, depression, and quality of life in adults with moderate-to-severe AD, while secukinumab treatment in psoriasis was associated with improved patient perception of anxiety and depression (90, 91). In PN, phase 3 trials showed that dupilumab significantly improved severe pruritus and skin lesions, and nemolizumab also reduced the signs and symptoms of disease (92, 93). These findings suggest that targeted biologics may reduce part of the emotional burden associated with chronic pruritus, but they should be integrated with psychological and behavioral care when emotional distress, sleep disturbance, or compulsive scratching remains clinically relevant.

#### 4.2.5 Neurobiological mechanisms from a skin-brain axis perspective

The bibliometric findings suggest that mechanistic interpretation has become an increasingly visible direction in this field. Keyword and co-cited reference analyses showed growing attention to inflammation, oxidative stress, mechanisms, mental health, amygdala, and pruritus receptor units, indicating increasing interest in both central affective circuits and peripheral pruritus signaling. From this perspective, the skin-brain axis provides an integrative framework to understand the bidirectional interactions between the brain, peripheral nerves, skin, neuroendocrine pathways, and immune mediators: chronic pruritus may lead to anxiety and depression symptoms through skin-to-brain signaling, while negative emotional states may in turn exacerbate pruritus through brain-to-skin neuroendocrine and neuroimmune pathways (5, 24, 94). Because anxiety and depression are the most prominent negative emotions in the two analyses, they are the main focus of the following discussion.

Within this skin-brain axis framework, human neuroimaging studies provide objective evidence that chronic pruritus involves central brain networks. Structural magnetic resonance imaging (MRI) and fMRI studies suggest that pruritus processing is not confined to somatosensory pathways, but also involves central networks related to affective processing, intrinsic brain activity, and stress regulation. fMRI evidence has identified an amygdala network for human pruritus processing, linking pruritus perception with negative affect and emotional memory (95). Evidence from resting state MRI further suggests that chronic pruritus severity is associated with the right default mode network, indicating that persistent pruritus may involve intrinsic brain network activity rather than peripheral sensory input alone (96). Stress-related central regulation is another key component of this framework. Structural MRI evidence has linked lower hypothalamic volume with higher perceived stress in patients with AD, suggesting that neuroendocrine stress regulation may be involved in the pruritus-emotion cycle (97). This interpretation is supported by fMRI evidence from neurodermatitis, which indicates that stress-induced pruritus is associated with central pathways for stress processing (98). Overall, these studies add a human neuroimaging basis to the skin-brain axis framework, showing that chronic pruritus is linked to central affective processing, intrinsic brain activity, and stress regulation.

Pruritus is not only a sensory symptom, but also an emotional experience (99). Chronic pruritus engages brain regions involved in emotional and motivational processing, including the insula, amygdala and anterior cingulate cortex, which helps to explain why persistent pruritus is closely related to anxiety symptoms (100). Experimental studies further suggest that central circuits are involved in the affective response to pruritus. For instance, lateral septum γ-aminobutyric acid neurons and chloroquine-sensitive neurons in the central amygdala have been identified as critical factors in regulating the pruritus-anxiety cycle (101, 102). Repeated scratching caused by pruritus can further damage the skin barrier, exacerbate local skin inflammation, and promote the release of neuropeptides, cytokines, and other mediators, thereby maintaining local sensitivity and sustained skin-to-brain input (103, 104). Anxiety symptoms are often accompanied by heightened perceived stress and physiological arousal (24, 105). Consistent with the neuroimaging evidence on central stress regulation, stress-related activation of the HPA axis and autonomic nervous system may partly explain how anxiety amplifies pruritus perception (24, 105). In this brain-to-skin pathway, corticotropin-releasing hormone (CRH) and glucocorticoid signaling may modulate mast cell and keratinocyte activity and inflammatory cytokine signaling, while interleukin-31 (IL-31) contributes to neuronal sensitization and persistent pruritus (103, 105, 106).

A similar bidirectional pattern can also be observed in depressive symptoms, particularly when pruritus becomes chronic, persistent, and difficult to control. Under these conditions, the burden of repeated pruritus may extend beyond the skin and gradually interfere with the emotional regulation function of the central system through sustained neuroendocrine dysregulation and persistent neuroimmune signals (103, 104). Experimental evidence has shown that chronic pruritus can affect emotion and HPA axis function, providing a neuroendocrine basis for the relationship between pruritus and depressive symptoms (17). Behavioral evidence also supports a bidirectional interaction. Chronic pruritus is associated with more depressive-like behavior, while depressive-like states are accompanied by increased spontaneous scratching and stronger responses to pruritus stimuli (107). Based on these behavioral findings, a recent viewpoint has proposed a “itch-to-brain circuitry” model in AD, indicating that persistent pruritus may gradually alter the central pathway involved in depressive symptoms (108). Conversely, depressive symptoms may also aggravate pruritus through prolonged stress burden and persistent neuroendocrine dysregulation (24, 109). When depressive symptoms persist, stress-related changes in CRH and glucocorticoid activity may enhance skin neuroendocrine and immune responses, thereby promoting the release of pruritus mediators, neuronal sensitization and persistent pruritus (105, 109).

Together, anxiety and depressive symptoms should not be regarded merely as psychological consequences of chronic pruritus. Rather, they may participate in reciprocal skin-to-brain and brain-to-skin processes involving central affective circuits, stress-related neuroendocrine activity, and neuroimmune signaling.

## 5 Advantages and limitations

To our knowledge, this study is the first bibliometric and visual analysis to explore the bidirectional connections between pruritus and negative emotions. By employing multiple complementary bibliometric and visualization tools (VOSviewer, CiteSpace, Charticulator, and Bibliometrix) and supplementing WoSCC data with PubMed clinical trials, this study systematically maps research evolution, major trends, and emerging priorities in this interdisciplinary area.

Despite these methodological strengths, several limitations must be acknowledged. First, the analysis was limited to WoSCC and PubMed. Although these databases provide compatible citation metadata and clinical trial coverage, excluding Scopus, Embase, PsycINFO, and CINAHL may have missed relevant psychological, psychiatric, psychosomatic, nursing, and allied health studies. Second, despite the broad search strategy, some records using alternative concepts or discipline specific terminology may not have been retrieved. Third, keyword standardization reduced term fragmentation but may have influenced keyword frequencies, co-occurrence patterns, and cluster structure. Fourth, citation indicators were based on raw cumulative counts and were not normalized by publication year, which may introduce temporal bias and affect the interpretation of research hotspots and thematic evolution. Fifth, the supplementary PubMed clinical trial analysis included relatively few eligible trials and was designed to describe intervention patterns, not to compare treatment effects or infer clinical superiority.

## 6 Conclusion

This study conducted a systematic bibliometric and visual analysis of the global research status of pruritus and negative emotions from 1980 to 2026. The United States maintains a preeminent position in publication output, citation influence, and international cooperation. Overall, our findings delineate a distinct trajectory: the field has evolved from merely describing clinical burden and emotional comorbidities toward a more multidimensional, mechanistic research agenda.

Anxiety and depression remain the dominant emotional constructs, while patient-reported outcomes, sleep disturbance, cognitive processes, and neurobiological mechanisms have received increasing attention. From a mechanistic perspective, the skin-brain axis provides a useful framework for interpreting the reciprocal relationship between chronic pruritus and negative emotions. In clinical intervention research, targeted biologics have become highly visible, particularly in inflammatory pruritic skin diseases, but their prominence should be interpreted alongside the need for psychological, behavioral, and psychodermatological care. Future research should move beyond pruritus intensity alone and further integrate multidimensional PROMs, emotional and cognitive processes, skin-brain axis mechanisms, and interventions that address both pruritus and emotional burden.
