# Supplementary material for: Breaking the vicious cycle: a bibliometric and visual analysis of the bidirectional relationship between pruritus and negative emotions
Source: Front Psychiatry. 2026 Jul 30;17:1883590. doi: 10.3389/fpsyt.2026.1883590 (PMC13471740; doi:10.3389/fpsyt.2026.1883590)
Supplement: Supplementary file 1 [file SupplementaryFile1.docx]

**Table S1 Threshold settings and number of nodes before and after thresholding in VOSviewer analyses**

| **Network analysis** | **Threshold** | **Nodes before thresholding** | **Nodes after thresholding** |
| --- | --- | --- | --- |
| Institutional collaboration | ≥13 publications | 3634 | 57 |
| Author collaboration | ≥7 publications | 13098 | 40 |
| Author co-citation | ≥42 citations | 61113 | 122 |
| WoSCC keyword co-occurrence | ≥31 occurrences | 9303 | 93 |
| PubMed keyword co-occurrence | ≥4 occurrences | 204 | 32 |

**Table S2 Thesaurus terms used for keyword standardization in VOSviewer.**

| **Label** | **Replace by** | **Label** | **Replace by** |
| --- | --- | --- | --- |
| itching | pruritus | PROMs | patient-reported outcomes |
| itch | pruritus | patient reported outcome | patient-reported outcomes |
| pruritis | pruritus | patient-reported outcome | patient-reported outcomes |
| pruritic sensation | pruritus | patient reported outcomes | patient-reported outcomes |
| pruritic symptom | pruritus | Patient-reported outcome measure | patient-reported outcomes |
| chronic itch | chronic pruritus | Patient-reported outcome measures | patient-reported outcomes |
| chronic itching | chronic pruritus | antibodies, monoclonal, humanized | antibodies, monoclonal |
| chronic pruritis | chronic pruritus | antibodies, monoclonal | antibodies, monoclonal |
| health-related quality of life | quality of life | depressive symptoms | depression |
| QoL | quality of life | anxiety symptoms | anxiety |
| quality-of-life | quality of life | chronic kidney-disease | chronic kidney disease |
| atopic eczema | atopic dermatitis | CKD | chronic kidney disease |
| atopic-dermatitis | atopic dermatitis | nodular prurigo | prurigo nodularis |
| dermatitis, atopic | atopic dermatitis | prurigo, nodular | prurigo nodularis |
| patient reported outcome measures | patient-reported outcomes | PN | prurigo nodularis |
| patient reported outcome measure | patient-reported outcomes | DLQI | Dermatology Life Quality Index |
| PROs | patient-reported outcomes | dermatology life quality index | Dermatology Life Quality Index |

**Table S3 Annual number of WoSCC publications on pruritus and negative emotions from 1980 to 2025.**

| **Year** | **Number of annual publications** | **Year** | **Number of annual publications** |
| --- | --- | --- | --- |
| 1980 | 1 | 2003 | 24 |
| 1981 | 3 | 2004 | 25 |
| 1982 | 2 | 2005 | 32 |
| 1983 | 4 | 2006 | 42 |
| 1984 | 5 | 2007 | 37 |
| 1985 | 7 | 2008 | 46 |
| 1986 | 7 | 2009 | 44 |
| 1987 | 10 | 2010 | 64 |
| 1988 | 6 | 2011 | 46 |
| 1989 | 14 | 2012 | 72 |
| 1990 | 15 | 2013 | 66 |
| 1991 | 21 | 2014 | 60 |
| 1992 | 17 | 2015 | 62 |
| 1993 | 19 | 2016 | 89 |
| 1994 | 29 | 2017 | 99 |
| 1995 | 20 | 2018 | 111 |
| 1996 | 19 | 2019 | 112 |
| 1997 | 21 | 2020 | 141 |
| 1998 | 25 | 2021 | 169 |
| 1999 | 28 | 2022 | 155 |
| 2000 | 20 | 2023 | 185 |
| 2001 | 20 | 2024 | 183 |
| 2002 | 30 | 2025 | 240 |

**Table S4 Cluster summary and thematic interpretation of the WoSCC keyword co-occurrence network**

| **Cluster** | **Cluster label** | **Number of keywords** | **Top 10 keywords** | **Interpretation** | **Representative references** |
| --- | --- | --- | --- | --- | --- |
| Cluster 1 | Clinical burden, assessment, and psychosocial outcomes | 36 | quality of life, depression, prevalence, psoriasis, anxiety, impact, disease, eczema, symptoms, health | This cluster reflects the clinical and psychosocial burden of pruritus, especially its association with anxiety, depression, sleep disturbance, quality of life, and patient-reported assessment. | Finlay and Khan, 1994 (1); Zigmond and Snaith, 1983 (2); Gupta et al., 1994 (3); Schneider et al., 2006 (4) |
| Cluster 2 | Pain management and opioid associated pruritus | 26 | pain, management, double-blind, efficacy, children, morphine, analgesia, postoperative pain, fentanyl, bupivacaine | This cluster mainly reflects perioperative pain management and analgesic contexts of pruritus, especially opioid-associated pruritus related to morphine, anesthesia, and postoperative pain. | Kumar and Singh, 2013 (5); Becker et al., 2022 (6); Jannuzzi, 2016 (7) |
| Cluster 3 | Inflammatory and neurobiological mechanisms | 18 | pruritus, atopic dermatitis, stress, skin, oxidative stress, inflammation, expression, mechanisms, activation, cells | This cluster represents the mechanistic frontier of the field, linking inflammatory signaling, neuroimmune pathways, stress-related mechanisms, and pruritus. | Cevikbas and Lerner, 2020 (8); Cevikbas et al., 2014 (9); Zhao et al., 2018 (10); Sanders et al., 2019 (11) |
| Cluster 4 | Chronic kidney disease associated pruritus | 7 | uremic pruritus, chronic kidney disease, outcomes, hemodialysis-patients, hemodialysis, dialysis, fatigue | This cluster focuses on chronic kidney disease-associated pruritus, particularly in dialysis and hemodialysis populations, and highlights its association with fatigue, clinical outcomes, and patient burden. | Fishbane et al., 2020 (12); Bai et al., 2025 (13); Poku et al., 2022 (14) |
| Cluster 5 | Targeted therapy and clinical care | 6 | therapy, adults, placebo, moderate, care, dupilumab | This cluster reflects therapeutic evaluation and clinical care, particularly the emergence of targeted therapies for moderate-to-severe pruritic diseases. | Cork et al., 2020 (15); Yosipovitch et al., 2023 (16); Kwatra et al., 2023 (17) |

*****Keywords are listed according to occurrence frequency. For clusters containing fewer than ten keywords, all keywords in the cluster are reported.

**References**

1.	Finlay AY, Khan GK. Dermatology Life Quality Index (DLQI)-a simple practical measure for routine clinical use. *Clin Exp Dermatol* (1994) 19:210–216. doi: 10.1111/j.1365-2230.1994.tb01167.x

2.	Zigmond AS, Snaith RP. The Hospital Anxiety and Depression Scale. *Acta Psychiatr Scand* (1983) 67:361–370. doi: 10.1111/j.1600-0447.1983.tb09716.x

3.	Gupta MA, Gupta AK, Schork NJ, Ellis CN. Depression modulates pruritus perception: a study of pruritus in psoriasis, atopic dermatitis, and chronic idiopathic urticaria.: *Psychosom Med* (1994) 56:36–40. doi: 10.1097/00006842-199401000-00005

4.	Schneider G, Driesch G, Heuft G, Evers S, Luger TA, Ständer S. Psychosomatic cofactors and psychiatric comorbidity in patients with chronic itch. *Clin Exp Dermatol* (2006) 31:762–767. doi: 10.1111/j.1365-2230.2006.02211.x

5.	Kumar K, Singh SI. Neuraxial opioid-induced pruritus: An update. *J Anaesthesiol Clin Pharmacol* (2013) 29:303–307. doi: 10.4103/0970-9185.117045

6.	Becker LM, Teunissen AJW, Koopman JSHA. Prevention and Treatment of Neuraxial Morphine-Induced Pruritus: A Scoping Review. *J Pain Res* (2022) 15:1633–1645. doi: 10.2147/JPR.S361225

7.	Jannuzzi RG. Nalbuphine for Treatment of Opioid-induced Pruritus A Systematic Review of Literature. *Clin J Pain* (2016) 32:87–93. doi: 10.1097/AJP.0000000000000211

8.	Cevikbas F, Lerner EA. Physiology and Pathophysiology of Itch. *Physiol Rev* (2020) 100:945–982. doi: 10.1152/physrev.00017.2019

9.	Cevikbas F, Wang X, Akiyama T, Kempkes C, Savinko T, Antal A, et al. A sensory neuron-expressed IL-31 receptor mediates T helper cell-dependent itch: Involvement of TRPV1 and TRPA1. *J Allergy Clin Immunol* (2014) 133:448–460. doi: 10.1016/j.jaci.2013.10.048

10. Zhao X, Yu C, Ye F, Wang Y-G, Mei Q-Y, Ma Q, et al. Chronic itch impairs mood and HPA axis function in mice: modulation by CRFR1 antagonist. *PAIN* (2018) 159:2201–2213. doi: 10.1097/j.pain.0000000000001319

11.	Sanders KM, Sakai K, Henry TD, Hashimoto T, Akiyama T. A Subpopulation of Amygdala Neurons Mediates the Affective Component of Itch. *J Neurosci* (2019) 39:3345–3356. doi: 10.1523/JNEUROSCI.2759-18.2019

12. Fishbane S, Jamal A, Munera C, Wen W, Menzaghi F. A Phase 3 Trial of Difelikefalin in Hemodialysis Patients with Pruritus. *N Engl J Med* (2020) 382:222–232. doi: 10.1056/NEJMoa1912770

13. Bai L, Shi J, Du X. Patient-Reported Outcome Measures in CKD-Associated Pruritus: A Systematic Review. *Kidney Med* (2025) 7:101055. doi: 10.1016/j.xkme.2025.101055

14. Poku E, Harnan S, Rooney G, Martyn-St James M, Hernandez-Alava M, Schaufler T, et al. The relationship between chronic kidney disease-associated pruritus and health-related quality of life: a systematic review. *Clin Kidney J* (2022) 15:484–499. doi: 10.1093/ckj/sfab218

15. Cork MJ, Eckert L, Simpson EL, Armstrong A, Barbarot S, Puig L, et al. Dupilumab improves patient-reported symptoms of atopic dermatitis, symptoms of anxiety and depression, and health-related quality of life in moderate-to-severe atopic dermatitis: analysis of pooled data from the randomized trials SOLO 1 and SOLO 2. *J Dermatol Treat* (2020) 31:606–614. doi: 10.1080/09546634.2019.1612836

16. Yosipovitch G, Mollanazar N, Ständer S, Kwatra SG, Kim BS, Laws E, et al. Dupilumab in patients with prurigo nodularis: two randomized, double-blind, placebo-controlled phase 3 trials. *Nat Med* (2023) 29:1180–1190. doi: 10.1038/s41591-023-02320-9

17. Kwatra SG, Yosipovitch G, Legat FJ, Reich A, Paul C, Simon D, et al. Phase 3 Trial of Nemolizumab in Patients with Prurigo Nodularis. *N Engl J Med* (2023) 389:1579–1589. doi: 10.1056/NEJMoa2301333
